# Effectors Targeting the Unfolded Protein Response during Intracellular Bacterial Infection

**DOI:** 10.3390/microorganisms9040705

**Published:** 2021-03-29

**Authors:** Manal H. Alshareef, Elizabeth L. Hartland, Kathleen McCaffrey

**Affiliations:** 1 Centre for Innate Immunity and Infectious Diseases, Hudson Institute of Medical Research, Clayton, VIC 3168, Australia; manal.alshareef@monash.edu; 2 Department of Molecular and Translational Science, Monash University, Clayton, VIC 3168, Australia; 3 College of Pharmacy, Umm Al-Qura University, Makkah 24230, Saudi Arabia

**Keywords:** ER stress, UPR, bacteria, infection, secretion systems, effector proteins

## Abstract

The unfolded protein response (UPR) is a homeostatic response to endoplasmic reticulum (ER) stress within eukaryotic cells. The UPR initiates transcriptional and post-transcriptional programs to resolve ER stress; or, if ER stress is severe or prolonged, initiates apoptosis. ER stress is a common feature of bacterial infection although the role of the UPR in host defense is only beginning to be understood. While the UPR is important for host defense against pore-forming toxins produced by some bacteria, other bacterial effector proteins hijack the UPR through the activity of translocated effector proteins that facilitate intracellular survival and proliferation. UPR-mediated apoptosis can limit bacterial replication but also often contributes to tissue damage and disease. Here, we discuss the dual nature of the UPR during infection and the implications of UPR activation or inhibition for inflammation and immunity as illustrated by different bacterial pathogens.

## 1. Introduction

### 1.1. Endoplasmic Reticulum Stress and the Unfolded Protein Response

The endoplasmic reticulum (ER) is the entry point to the secretory pathway and approximately one-third of proteins within the cell are biosynthesized at the ER [1]. Sites of protein synthesis, or the “rough” ER, are enriched for membrane-associated ribosomes which translocate nascent proteins into the ER lumen and insert integral membrane proteins into the ER membrane. In the ER, proteins undergo oxidative folding as assisted by ER chaperones and acquire unique post-translational modifications, such as asparagine (N)-linked glycosylation, which are often essential for their function. After correct folding, proteins are released from ER exit sites (ERES), or the “smooth” ER, and undergo vesicle transport through the secretory pathway to the plasma membrane, extracellular space or other organelles within the endomembrane system. In addition to protein biosynthesis, lipid and cholesterol biosynthesis occur at the ER and the ER is the major calcium storage organelle within the cell.

**Figure 1:**
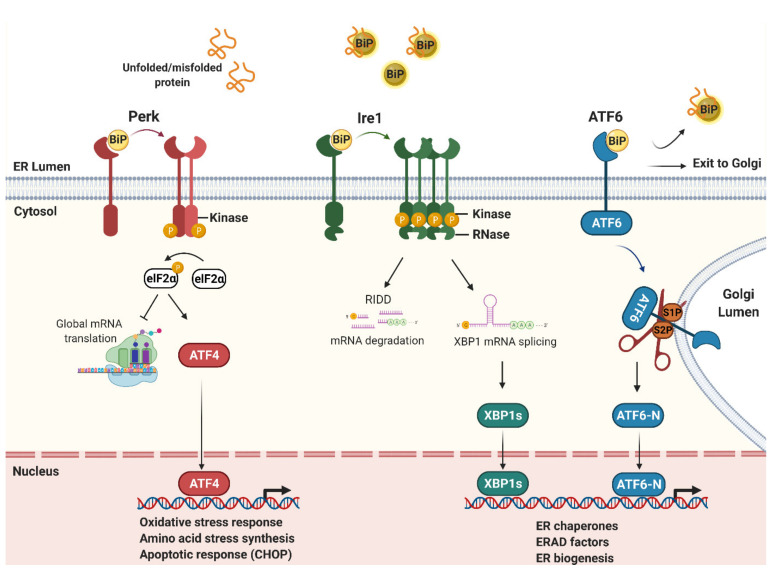
ER stress and the unfolded protein response (UPR). The UPR is an intracellular signaling cascade activated by misfolded proteins within the ER lumen. Misfolded proteins sequester BiP to release Perk, Ire1 and ATF6 to initiate downstream signaling. Perk is a kinase which phosphorylates the translation initiation factor eIF2α to repress protein synthesis, but selectively increases the translation of ATF4 mRNA via upstream open reading frames (uORFs) in its 5′ untranslated region (UTR). ATF4 is a transcription factor which increases the transcription of the pro-apoptotic factor CHOP. Ire1 is a bi-functional kinase/endoribonuclease (RNase) which splices XBP1 mRNA in the cytoplasm to generate the active transcription factor, XBP1s. Ire1 also cleaves other ER-targeted mRNAs which are then degraded, a process called regulated Ire1-dependent degradation (RIDD). ATF6 is cleaved by site-1 (S1P) and site-2 (S2P) proteases in the Golgi to release its N-terminal transcription factor domain (ATF6-N) that translocates into the nucleus. The single or combined action of XBP1s and ATF6-N up-regulates the transcription of many ER stress-responsive genes to increase ER protein-folding and secretory capacity and to remove misfolded proteins via ER-associated degradation (ERAD).

ER stress occurs upon a loss of homeostasis which results in the accumulation of misfolded proteins in the ER (Figure 1). ER stress is caused by genetic disorders which cause the persistent misfolding of proteins in the ER but also various physiological or environmental challenges to the cell, including pathogen infection. As misfolded proteins overwhelm the protein-quality control machinery of the ER, the unfolded protein response (UPR) is activated [2]. The UPR is an intracellular signaling cascade which initiates various transcriptional and post-transcriptional programs to restore ER homeostasis (Figure 1). However, if ER stress is severe or prolonged, the UPR initiates apoptosis and is therefore often associated with tissue damage and disease [3].

In addition to infection, certain types of autoimmune and genetic diseases are associated with activation of the UPR, which is termed sterile ER stress [4]. In mammalian systems, sterile and non-sterile UPR signaling is regulated by three transmembrane proteins located at the ER membrane; protein kinase R (PKR)-like endoplasmic reticulum kinase (Perk/*EIF2AK3*) [5], inositol-requiring kinase 1 (Ire1/*ERN1*) [6], and activating transcription factor 6 α (ATF6/*ATF6*) [7]. Each protein has a stress-sensing domain, which detects misfolded proteins within the ER lumen, and a cytoplasmic signaling domain. Under normal conditions, Ire1, Perk and ATF6 are negatively regulated through interactions with the major ER Hsp70 chaperone BiP [8,9]. During ER stress, BiP is recruited to misfolded proteins within the ER lumen and therefore releases Ire1, Perk and ATF6 to initiate downstream signaling. As described below, the UPR combats ER stress by various mechanisms including inhibiting protein biosynthesis at the ER, expanding ER protein-folding capacity or removing misfolded proteins from the ER.

### 1.2. The ER Stress Sensors: Ire1, Perk and ATF6

Ire1 is bi-functional kinase and an endoribonuclease (RNase) which dimerizes and is auto-phosphorylated via its kinase domain to form larger signaling complexes during ER stress [10,11]. The Ire1 RNase domain unconventionally splices *XBP1* mRNA which, after re-ligation, is translated to produce the active XBP1 bZIP transcription factor, XBP1s [12]. In mammalian systems, XBP1s upregulates a number of ER stress responsive genes including key factors required for dislocating misfolded proteins from the ER for proteasomal degradation in the cytosol, a process called ER-associated degradation (ERAD) [13]. Ire1 also cleaves a number of other ER-targeted mRNAs, a process called regulated Ire1-dependent decay (RIDD) [14], which helps reduce the number of proteins entering the ER during ER stress.

Perk is a protein kinase which, when activated due to ER stress, dimerizes and auto-phosphorylates via its cytoplasmic kinase domain [5]. Once active, Perk phosphorylates eukaryotic translation initiation factor-α (eIF2α) which inhibits translation initiation and therefore global protein synthesis to reduce nascent proteins entering the ER [15]. Several mRNAs are exempt from translational repression, including the transcription factor ATF4, which contains regulatory elements within its 5′ UTR that selectively increase translation during ER stress [16]. ATF4 drives the expression of ER stress responsive genes required for amino-acid and oxidative stress resistance, including CHOP which initiates apoptosis in response to severe stress [3].

ATF6 is a membrane-associated transcription factor which, during ER stress, traffics to the Golgi apparatus where it is cleaved by site 1 (S1P) and site 2 (S2P) proteases to release a cytosolic N-terminal bZIP transcription factor domain (ATF6-N) [7]. ATF6-N is translocated into the nucleus to upregulate many ER stress responsive genes and, in mammalian systems, ATF6-N is required for the increased production of all major ER chaperones, including BiP. ATF6 is therefore necessary for increasing ER protein-folding and secretory capacity during ER stress to restore ER homeostasis [13,17,18].

### 1.3. The UPR and Impact on Immunity and Intracellular Bacterial Infection

**Figure 2:**
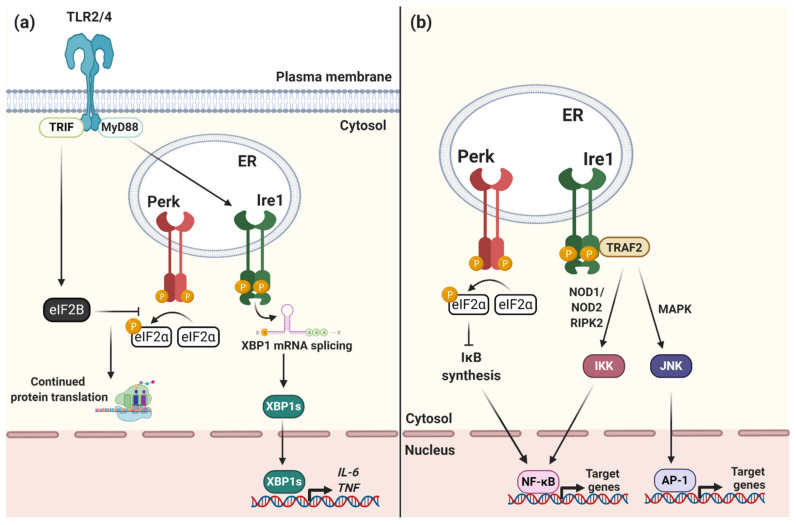
The UPR drives pro-inflammatory responses to ER stress. (**a**) TLR signaling activates Ire1 and XBP1 mRNA splicing via a MyD88-dependent pathway. Translocation of XBP1s into the nucleus enhances the transcription of pro-inflammatory cytokines including IL-6 and TNFα. TLR signaling via TRIF activates eIF2B GEF activity to counteract the activity of phosphorylated eIF2α. This allows protein synthesis, and therefore cytokine production, to proceed despite Perk activation and delays pro-apoptotic signaling via CHOP. (**b**) Ire1 interacts with TRAF2 to activate IKK and JNK signaling cascades. IKK and JNK activate the pro-inflammatory transcription factors NF-κB and AP-1, respectively, to drive the production of pro-inflammatory cytokines during ER stress. Perk signaling and eIF2α phosphorylation inhibits biosynthesis of inhibitor of κB (IκB) which increases NF-κB transcriptional activity and pro-inflammatory cytokine production.

Although the UPR is primarily a homeostatic response to ER stress, it also has an important role in immunity. The UPR is essential for the function of many immune cell types which, during infection, require the rapid expansion of their ER to produce antibodies, cytokines or other immune factors [19]. In macrophages, for example, Toll-like receptor (TLR) signaling directly regulates the UPR in order to enhance pro-inflammatory cytokine production during infection [20] as well as to prolong the life of these cells despite severe ER stress [21] (Figure 2a). TLR signaling via TRIF stimulates eIF2B GEF activity to counteract the activity of phosphorylated eIF2α. This allows protein synthesis, and therefore cytokine production, to proceed despite Perk activation and delays pro-apoptotic signaling via CHOP [21]. Rapamycin, which inhibits cap-dependent translation via mTOR, has also been shown to stimulate innate immune signaling during bacterial infection by selectively increasing the translation of pro-inflammatory cytokines [22], although it remains unclear how exactly these transcripts are exempt from translational inhibition and is an exciting area for further research [23].

The UPR also leads to activation of the transcription factors NF-κB and AP-1 to increase pro-inflammatory cytokine production during ER stress [24,25,26], whether caused by infection or other sterile forms of cell stress, such as protein misfolding or oxidative stress, further indicating that the UPR is part of the pro-inflammatory response [27] (Figure 2b). Ire1 interacts with TRAF2 to activate NF-κB and AP-1 thereby increasing TNF and IL-6 production [24,25]. Global protein translation inhibition resulting from PERK activation and eIF2α phosphorylation can also lead to prolonged activation of NF-κB [28]. This prolonged activation is due to sustained loss of IκB and A20, both NF-κB inhibitors, due to disrupted protein synthesis. When NF-κB is activated for a prolonged period this selectively enhances the transcription of a subset of NF-κB target genes, including *Il23a* and *Csf2* [29]. Sustained innate immune responses to translation inhibition show the complexity of post-transcriptional regulation of cytokine production during ER-stress.

Given its influence on host cell survival and the inflammatory response, ER stress is a common feature of many bacterial infections [30]. ER stress can be caused by bacterial virulence factors that directly disrupt the ER and its secretory functions or ER stress can be triggered indirectly, due to the depletion of nutrients and other cofactors required for ER function. For example, intracellular bacterial pathogens that are adapted to replicate within a eukaryotic host and derive nutrients from the host cell will likely activate ER stress.

Emerging evidence suggests that some bacterial pathogens directly target the UPR during infection through the activity of secreted effector proteins and toxins. Hence, this may be a common strategy shared by different pathogens to promote bacterial replication and/or evade host immunity (Table 1). Here, we discuss the different mechanisms utilized by a number of key bacterial pathogens to manage ER stress during infection and the emerging role of the UPR in host responses to these pathogens.

### 1.4. Activation of the UPR by Brucella

*Brucella* is a highly contagious, Gram-negative bacterium which causes abortion and sterility in cattle as well as other agricultural animals. It can also be transmitted to humans to cause a recurrent fever as well as other more serious neurological complications. The most common species associated with human disease are *Brucella melitensis*, *B. abortus*, *B. canis*, and *B. suis*. *Brucella* is an intracellular bacterial pathogen which replicates in phagocytic cells, including macrophages and dendritic cells, as well as placental trophoblasts.

After bacterial uptake by the host cell, *Brucella* resides within a membrane-bound compartment which matures along the endocytic pathway and partially fuses with lysosomes to form an acidified compartment. This step is essential for activation of the bacterial VirB type-IV secretion system (T4SS) [53]. The VirB T4SS is required to translocate bacterial virulence factors across the bacterial membrane into the host cell. These effector proteins regulate various host cell processes required for the formation of a replication vacuole within the host cell, termed the *Brucella*-containing vacuole (BCV) [54]. Maturation of the BCV requires extensive interactions with ER exit sites (ERES) and this dramatic restructuring of ER tubules during *Brucella* infection causes ER stress and activation of the UPR [32,55]. RNAi knockdown of Ire1 however reduces *Brucella*’s ability to replicate, suggesting that the UPR has a direct role in promoting bacterial replication [56]. A subsequent study showed that Ire1 phosphorylates ULK1 (Atg1), an autophagy initiation factor, which promotes ER-derived autophagy from ERES and is therefore important for maturation of the replication-permissive BCV during *Brucella* infection [57].

During infection, the VirB T4SS effector protein VceC localizes to the ER membrane where it binds BiP to induce ER stress, also suggesting that activation of the UPR is favourable for *Brucella* replication [31]. Another VirB T4SS effector protein, TcpB, which is involved in restructuring of ER tubules, has also been shown to activate the UPR and several other *Brucella* effectors, including BspC, BspG, BspH and BspK, induce ER stress although their functions have not yet been characterized [33]. TLR-dependent activation of Ire1 in macrophages also enhances *Brucella* replication in a MyD88-dependent manner [32] (Figure 2a)

Activation of the UPR, and specifically Ire1, initiates pro-inflammatory signaling via interactions with TRAF2 (Figure 2b) and *Brucella* infection drives NOD1- and NOD2-dependent cytokine production in a VceC- and ER-stress dependent manner [26]. The UPR therefore contributes to acute inflammation during *Brucella* infection although it does not appear to restrict bacterial replication in vivo. In placental trophoblasts, VceC-induced ER stress was also shown to contribute to abortions in pregnant mice via activation of Perk and downstream pro-apoptotic signaling via the transcription factor CHOP [58]. Together, this suggests that *Brucella* infection drives ER stress and UPR signaling to promote bacterial replication, but that UPR-mediated inflammation and apoptosis also contribute to disease.

### 1.5. Inhibition of the UPR by Legionella

*Legionella* is a Gram-negative, bacterial pathogen found in soil and water environments that replicates within predatory protists, including amoebae [59]. *Legionella* opportunistically infects human alveolar macrophages after the inhalation of contaminated water aerosols which can result in a life-threatening form of pneumonia called Legionnaire’s disease. The most common *Legionella* species associated with human disease are *L. pneumophila* and *L. longbeachae*. After phagocytosis, *Legionella* resides within a membrane-bound compartment and hijacks ER vesicles destined for the Golgi to establish a replicative vacuole, termed the *Legionella* containing vacuole (LCV) [60]. Biogenesis of the LCV requires the bacterial Dot/Icm T4SS secretion system that translocates more than 300 effector proteins into the infected host cell. The resulting mature LCV is therefore a unique ER-like compartment, studded with membrane-associated ribosomes and enriched for many ER chaperones and other protein-folding factors as well as Dot/Icm effector proteins.

Surprisingly, despite hijacking the ER, UPR signaling is not observed during *Legionella* infection and two independent studies have shown that *Legionella* inhibits *XBP1* mRNA splicing in infected macrophages when challenged with chemically induced ER stress [34,35]. Both studies also identified the Dot/Icm effector proteins, Lgt1, Lgt2 or Lgt3, as necessary for this inhibition. The Lgt proteins are redundant glucosyltransferases that inhibit translation elongation during infection [61]. They therefore indirectly inhibit *XBP1* mRNA splicing caused by chemically induced ER stress [35]. However, *Legionella* mutants lacking the Lgt proteins do not show any defects in bacterial replication in vitro or in vivo [29] and therefore the role of the UPR during *Legionella* infection remains unclear. Heat-inactivated *Legionella* activates TLR-dependent *XBP1* mRNA splicing via a MyD88-dependent pathway (Figure 2a) but this was not observed during infection with live, replication-competent *Legionella* [35]. This suggests that the Lgt proteins may dampen UPR-dependent inflammatory responses to infection although this has not been directly investigated. Overall, this suggests that *Legionella* inhibits UPR signaling although the significance of this inhibition for bacterial replication, or host immune responses to infection, is yet to be determined.

### 1.6. Implications of Severe ER Stress for Mycobacterial Infection

*Mycobacterium tuberculosis* is the cause tuberculosis and an intracellular bacterial pathogen with a unique “myco-membrane” structure. *M. tuberculosis* infects alveolar macrophages and has an ESX-1 type-VII secretion system (T7SS) which is essential for inhibiting phagosome maturation and release of the bacteria into the host cytosol [62,63]. Although *M. tuberculosis* replicates within the host cytosol, it induces a severe ER stress response which drives apoptosis of the host cell [64]. *M. tuberculosis* infection dramatically increases intracellular Ca^2+^ and reactive oxygen species (ROS) which is likely to be the primary cause of ER stress. This is mediated, at least in part, by the T7SS protein, ESAT-6, which is essential for lysis of the phagosome, but which also increases intracellular Ca^2+^ and ROS production [36,37]. The T7SS effector protein, Rav0297, from the PE/PGS/PGRS protein family also localizes to the ER via its PGRS domain and increases intracellular Ca^2+^ and ROS to cause ER stress [38].

Consistent with severe ER stress, lung granulomas from *M. tuberculosis*-infected mice are highly enriched for the pro-apoptotic UPR transcription factor CHOP [65]. However, *M. tuberculosis* inhibits phosphorylation of eIF2α and silencing of CHOP increases bacterial replication [66]. This suggests that *M. tuberculosis* inhibits Perk signaling to delay apoptosis of the host cell thereby promoting bacterial replication. The Mpt64 protein (*Rv1980c*) has also been shown to localize to the ER to inhibit CHOP expression [39], although whether it inhibits upstream phosphorylation of Perk or eIF2α was not tested.

Severe ER stress caused by *M. tuberculosis* also results in non-canonical trafficking of the ER-resident chaperones BiP and calreticulin to be either secreted from the cell or localized to the plasma membrane, respectively [67,68]. These “leaky” chaperones therefore act as auto-antigens and pro-inflammatory signals during infection. Extracellular calreticulin, for example, was shown to bind the CXCR1/TNFR1 receptor complex to induce extrinsic apoptotic signaling and therefore reduce bacterial survival in macrophages independent of CHOP-mediated (intrinsic) apoptosis [68]. Together, this suggests that the UPR is a primarily protective response against *M. tuberculosis* infection which drives apoptosis to limit bacterial replication early during infection. However, apoptosis may also aid bacterial dissemination in the late stage of infection and contribute to disease.

### 1.7. Chlamydia and UPR-Mediated Inflammatory Signaling

*Chlamydia* is a Gram-negative, obligate intracellular bacterium which primarily infects epithelial cells. The major species responsible for human disease include *C. trachomatis,* which causes pelvic inflammation and infertility, and *C. pneumoniae*, which causes respiratory tract infections, including pneumonia. *Chlamydia* has a type-III secretion system (T3SS) which secretes effector proteins into the host cell to hijack various host-cell processes required to support bacterial replication, including host glucose and lipid metabolism. Within the host cell, *Chlamydia* replicates within a vacuole called the inclusion. Maturation of the inclusion requires the formation of synapses with the “rough” ER which facilitate the translocation of ER proteins into the inclusion [69]. The inclusion also interacts with ERES and chemical disruption of ERES function restricts bacterial replication [70].

The significant reorganization of the ER during *Chlamydia* infection results in ER stress and activation of the UPR both in vitro and in vivo [71]. Inhibition of Ire1 RNase activity or Perk kinase activity blocks formation of the *Chlamydia* inclusion during infection [72], suggesting that the UPR is required for bacterial survival and replication within the host cell. Although the mechanism by which the UPR promotes inclusion formation is unknown, Ire1 and Perk activation increases autophagy and other host metabolic functions important for bacterial replication. The T3SS effector proteins CT288 and Tarp also promote Ire1 oligomerization, and therefore downstream signaling, by driving assembly of the myosin-heavy chain complex II [40].

Activation of Ire1, however, drives pro-inflammatory cytokine production during *Chlamydia* infection [71,73]. As described for *Brucella* infection, NOD1/NOD2/RIPK2-dependent IL-6 cytokine production during *Chlamydia* infection is ER stress-dependent, presumably via the Ire1-TRAF2 pathway (Figure 2b) [71]. However, unlike *Brucella*, UPR-dependent inflammatory responses restrict *Chlamydia* replication, indicating a central role for the UPR in host defense against this pathogen. Hence, although activation of the UPR promotes *Chlamydia* inclusion formation, UPR-mediated inflammatory signaling helps restrict bacterial replication, illustrating the potentially dual functions of the UPR during bacterial infection.

### 1.8. Interactions of Salmonella with the UPR

*Salmonella enterica* is a Gram-negative intracellular bacterium which causes a range of gastrointestinal disease and typhoid fever. *Salmonella* uses two T3SSs to inject effector proteins into the host cell in order enter and to replicate intracellularly. During infection of epithelial cells, *Salmonella* activates the UPR which appears to enhance bacterial replication [41]. A recent study demonstrated that the T3SS effector protein, SlrP, localizes to the ER lumen to bind ERdj3, a Hsp40/DnaJ co-chaperone required for BiP function [42]. SlrP would therefore be predicted to drive the misfolding of proteins in the ER lumen and induce the UPR during infection, although this has not yet been directly investigated. Hence, the interaction of *Salmonella* with ER stress and UPR signalling and the consequences for the immune response require much more work.

### 1.9. Bacterial Toxins and ER Stress

Pore-forming toxins are produced by many bacterial pathogens. These toxins bind host-cell membranes and assemble into pores which permeabilize the membrane to ions, metabolites and proteins, thereby activating various host-cell stress responses, including ER stress. A loss of Ire1- or ATF6-dependent signaling can sensitize animals to pore-forming toxins suggesting that the UPR is important for host defense [74]. This protective role of the UPR is activated via a non-canonical p38 MAPK signaling pathway and therefore independent of ER stress.

*Listeria monocytogenes* secretes the pore-forming toxin, listeriolysin O (LLO), which is required for bacterial escape from the phagocytic compartment to enable bacterial replication within the host cytosol. LLO causes a loss of Ca^2+^ homeostasis within the host cell which induces ER stress and ER stress-dependent apoptosis [43]. Chemical induction of ER stress prior to *Listeria* infection restricts intracellular bacterial replication, consistent with a protective role for the UPR during *Listeria* infection. *Helicobacter* secretes the pore-forming toxin, VacA, which induces vacuole formation in host cells and also drives host-cell apoptosis. VacA-mediated apoptosis requires Perk-dependent pro-apoptotic signaling via CHOP. The induction of BiP and XBP1 splicing also strongly correlates with *Helicobacter*-induced gastric carcinogenesis, suggesting that the UPR may also play an important role in the pathogenesis of *Helicobacter* disease [44,75,76].

In addition to pore-forming toxins, the AB_5_ family of toxins produced by a number of bacterial pathogens also induce ER stress. AB toxins comprise a B-subunit required for cell binding and endocytosis and a non-covalently associated catalytic A-subunit, which targets host processes within the cell. The A-subunit of the subtilase AB toxin subfamily found in certain strains of *Escherichia coli* (STEC: Shiga-like toxin producing *Escherichia coli*) are serine proteases that specifically cleave BiP during infection and therefore drive protein misfolding, ER stress and activation of the UPR [47,48,49]. Another subfamily of AB_5_ toxins, including Cholera toxin and the Shiga-like toxins, undergo retrograde transport into the ER, where they interact with various components of the ERAD pathway to mediate retro-translocation of the catalytic subunit into the host-cell cytosol. Although these toxins do not cleave BiP, they do bind to BiP and other ER protein-folding factors during toxin unfolding within the ER [45,46,50,51], which activates the UPR and UPR-dependent apoptosis of the host cell [52,77].

## 2. Summary

The UPR clearly has a conflicting role in host defense against bacterial infection. Recent evidence that many secreted bacterial effector proteins directly target UPR signaling during infection suggests that the UPR is not simply a byproduct of infection but has a central role in the host response to these pathogens. Intracellular pathogens such as *Brucella* and *Chlamydia* require the UPR to form a replication vacuole within the host cell and therefore the UPR promotes the survival and proliferation of these pathogens. However, pro-inflammatory UPR signaling also restricts intracellular bacterial replication during *Chlamydia* infection and non-canonical activation of the UPR via the p38 MAPK pathway is a protective against bacterial pore-forming toxins. In addition, *Legionella* and *M. tuberculosis* both inhibit UPR signaling, potentially to delay ER stress-mediated inflammation and apoptosis whereas UPR-dependent inflammation and apoptosis during *Brucella* infection directly contributes to abortions in vivo. These contrasting examples all illustrate the potentially pathogenic role of the UPR in bacterial infection but also the potential to exploit the UPR to control pathogen replication. The recent development of small molecule agonists and antagonists of the UPR [78,79,80,81,82,83,84] will provide new tools to explore the role of UPR signaling during bacterial infection and define the mechanisms by which the UPR aids or combats bacterial disease.

## Figures and Tables

**Table 1:** Bacterial toxins and effector proteins that modulate the Unfolded Protein Response (UPR).

Bacterium	Toxin or Effector	Effect	Mode of Action	Reference
*Brucella*	VceC	Induces the UPR	Binds BiP	[31]
	TcpB (BtpA/Btp1]	Induces the UPR	Restructures ER tubules	[32]
	BspC, BspG, BspH, BspI, BspK	Induces the UPR	Unknown	[33]
*Legionella*	Lgt1, Lgt2, Lgt3	Inhibits XBP1 splicing	Inhibits translation elongation	[34,35]
*Mycobacteria*	ESAT-6, Heparin-Binding Haemagglutinin (HBHA)	Induces the UPR	Increases intracellular Ca^2+^ and Reactive Oxygen Species (ROS)	[36,37]
	Rv027	Induces the UPR	Increases intracellular Ca^2+^ and ROS	[38]
	Mpt64	Inhibits CHOP expression	Unknown, binds PIPs on ER membrane	[39]
*Chlamydia*	CT288, Tarp	Activates Ire1	Drives Ire1 oligomerisation	[40]
*Salmonella*	SlrP	Induces the UPR	Binds ERdj3, BiP cochaperone	[41,42]
*Listeria*	Listeriolysin O (LLO)	Induces the UPR	Increases intracellular Ca^2+^	[43]
*Helicobacter*	VacA	Activates Perk	Unknown	[44]
*Vibrio cholerae*	Cholera toxin (CT)	Induces the UPR	Binds BiP and ERdj3	[45,46]
*E. coli*	Subtilase cytotoxin	Induces the UPR	Cleaves BiP	[47,48,49]
	Shiga-like toxins (SLT)	Induces the UPR	Binds BiP and ERdj3	[50,51,52]

## Data Availability

Not applicable.
